# Hospital Complications and Frailty in Mexican Older Adults: An Emergency Care Cohort Analysis

**DOI:** 10.3389/fmed.2020.00505

**Published:** 2020-09-29

**Authors:** Mario Ulises Pérez-Zepeda, María Fernanda Carrillo-Vega, Olga Theou, Luis David Jácome-Maldonado, Carmen García-Peña

**Affiliations:** ^1^Dirección de Investigación, Instituto Nacional de Geriatría, México City, México; ^2^Department of Medicine, Dalhousie University, Halifax, NS, Canada; ^3^Centro de Investigación en Ciencias de la Salud (CICSA), FCS, Universidad Anáhuac México Campus Norte, Huixquilucan de Degollado, Mexico; ^4^School of Physiotherapy, Dalhousie University, Halifax, NS, Canada

**Keywords:** emergency department, delirium, physical functional performance, hospital complications, geriatric health services

## Abstract

**Objectives:** To describe the association of frailty level on admittance to the Emergency Department (ED) with various hospital complications including delirium, low phase angle, and low handgrip strength.

**Design:** Prospective cohort.

**Setting:** ED rooms of two public general hospitals in Mexico City.

**Participants:** A total of 548 persons 60 years or older who were admitted to the ED and who were alive during follow-up testing at home were included.

**Measurements:** A 32-item frailty index (FI) was measured on admission to the ED. Outcome measures included delirium, phase angle, and hand grip strength measured during different stages of the hospitalization (i.e., from admission to the ED through to follow-up at home).

**Results:** From this final sample, mean age was 76 years (± SD 7.2) and 58.4% (*n* = 320) were women. Mean waiting time in the ED was 5.1 h (± SD 6.2), the average stay in the ED was 99.9 (±68.2) h, and 274 subjects (50%) were admitted to a general ward after ED admission. FI was not associated with phase angle and was negatively associated with handgrip strength at admission to ED (β = −3.97, confidence interval [CI] 95% −5.56 −2.38, *p* < 0.001), discharge from ED (β = −3.94, CI 95% −5.97 −1.90, *p* < 0.001), and discharge from hospital (β = −4.93, CI 95% −7.68 −2.18, *p* = 0.01). FI was positively associated with delirium (β = 3.68, CI 95% 1.53–5.83, *p* < 0.01).

**Conclusion:** Higher frailty at ED admission was associated with lower hand grip strength and delirium during hospitalization in Mexican older adults.

## Introduction

Older adults have particular needs that are different from those of younger adults due to the accumulation of age-related health problems. This has been increasingly recognized by health professionals, along with the urgency to have systems in place that account for an aging population (1). In addition to the well-recognized and frequent chronic diseases in older adults, there are other specific conditions (e.g., geriatric syndromes, frailty, dementia) linked to aging with unique clinical features (2, 3). It is worth noting that acute health problems (e.g., elective surgery, decompensation of prevalent chronic diseases, traumatic injuries, etc.) are largely impacted by pre-existing problems that result in a complex health care process (4–6). Therefore, in addition to long-term care, other conventional health services are also widely used by older adults (e.g., acute, intensive, emergency care, coronary units) (7).

For many older adults, emergency care is merely a means to access further health services, something which can be an intricate process for individuals, especially when pertaining to health systems with limited specialized resources for geriatric care (8, 9). Furthermore, there is a trend toward making clinical decisions according only to age, and not to overall health (i.e., ageism) (10). Improvement of geriatric care in emergency departments (ED) begins by generating evidence that allows for tailored interventions that take into account the individual needs of older adults (11). Even though information on this matter has piled up in recent years (12); there are still some gaps to be filled, particularly in developing countries.

A well-recognized risk factor in old age –and relevant for geriatric care– is frailty. It has been characterized as a condition that renders an older adult vulnerable to stressors and the consequent development of a worse health status (13, 14). In fact, frailty –measured using diverse tools– has been shown to predict mortality in diverse settings, including acute care (15–18). Besides mortality, other adverse events have been shown to be more frequent in frail older adults (19–21). The use of frailty tools to discriminate those older adults with a higher risk of developing diverse outcomes has limited evidence in the ED setting. On the other hand, some of the so-called geriatric syndromes, such as delirium, incontinence –urinary and bowel–, depression, malnutrition, and others, are commonly presented as a complication of acute geriatric care (22). These conditions have been associated with frailty in other settings (23). Our study aims to describe the association of frailty level on admission to ED with hospital complications including delirium, low phase angle, and low handgrip strength in hospitalized Mexican older adults.

## Materials and Methods

### Design and Sample

We conducted secondary analysis of a Mexican ED cohort; complete objectives and procedures are available elsewhere (24). In summary, the main aim of the study was to test whether an intervention based on geriatric care training of emergency care resident physicians reduced frailty levels in patients 60 years or older who were admitted to the ED in one of two general hospitals in Mexico City. These hospitals are part of the largest health-social security system in the country, the Mexican Institute of Social Security-IMSS (*Instituto Mexicano del Seguro Social)*. This is a mandatory social security system that offers a comprehensive package of benefits to roughly half of the population in Mexico, including healthcare at all levels, as well as social and economic benefits (e.g., retirement pensions), covering the needs of non-governmental workers and their families. The system assigns each individual and immediate family member to a Family Medicine Unit (FMU), which includes the primary health care provider, with secondary and tertiary health care provided as needed based on referrals from the FMU.

Sample selection process was done consecutively, and participants were included if they were admitted to the ED after clinical evaluation performed by the hospital's physicians (triage—prior to ED admission). Patients with an imminent, acute, life-threatening condition that required immediate attention (intensive care), victims of a car accident, or patients who suffered second- or third-degree burns were excluded. For this secondary analysis, we only included individuals who were alive at the post-discharge home evaluation and with valid phase angle and frailty data (see below). All data included in this report was assessed by the research team, and not taken from the charts.

### Independent Variable: Frailty Index

To identify frailty levels at ED admission, we used the frailty index (FI), a widely used tool across settings including the ED (15). Using data gathered by the research team, 37 variables were screened to create the FI. The items were chosen from FI's published in previous Mexican population-based studies (e.g., Mexican Health and Aging Study) (25). Only 32 variables were included in the final FI; five variables were excluded because of a high number of missing values. Details about the FI items are provided in Supplementary Table 1. Construction and calculation of the FI was carried out according to standard procedures (26). Categorization of the FI was also done in order to facilitate comparisons between levels of frailty: <0.2, 0.2 to 0.39, 0.4 to 0.59, and ≥0.6. Furthermore, due to low sample size we only used two categories for multivariate analysis: <0.4 and ≥0.4.

### Outcome Variables: Complications

Delirium was assessed with a validated Spanish version of the Confusion Assessment Method (CAM) (27) and defined as a binary variable that distinguished between those who did not develop delirium during hospitalization, and those who developed delirium. Phase angle derived from bio-impedance analysis was included as a proxy of nutritional status and was measured with a body composition analyzer RJ L quantum IV. Measurements were taken in a supine position, with legs and arms opened at a 30° angle. Hand grip strength, a robust biomarker of health and a well-established physical performance test, was measured with a hydraulic dynamometer and reported in kilograms.

### Confounder Variables

Socio-demographic variables, such as age, sex, education, marital status, living condition, and self-rated financial situation, were included in analysis. Health-process variables of the first hospitalization were also included: waiting time (in hours) in the ED, length of stay (in hours) in the ED, total length of stay (in hours) at the hospital, and admission or not to the general/internal medicine ward. Other health variables included main diagnosis upon admission and number of reasons for admission. Finally, since this work is a secondary analysis of a clinical trial in which an educational intervention was tested, having received the intervention or not was also included as a co-variate.

### Procedures

Once admitted to the ED, patients were evaluated by trained nurses from the research team to determine whether they fulfilled the inclusion criteria. Written consent was then obtained, and baseline measurements were collected within the first two hours of admission (i.e., FI, confounder variables, etc.). The same research personnel monitored the status of the individuals (i.e., discharge from ED, transfer to a hospital ward, etc.).

Phase angle and hand grip strength were measured at four time-points: at admission to ED, at discharge from ED, at discharge from hospitalization, and at home 120 days after admission to the ED. Delirium status was collected at admission to ED, at discharge from ED, and at discharge from hospitalization.

### Analytic Plan

The descriptive analyses compared variables across the four frailty levels. If the variable was nominal or ordinal, a chi-squared test was performed; meanwhile, if the variable was qualitative, the Kruskal–Wallis test was used.

Ordinary least squares regressions were performed to test the relationships between phase angle and handgrip strength, and between frailty and the other independent variables. A regression was performed for each of the stages considered in this study: admission to ED, discharge from ED, discharge from hospitalization, and post-discharge at home evaluation. The independent variables employed were FI as a dichotomous variable (≤ 0.4), age, sex, marital status, schooling, living alone, self-rated financial situation, belonging to the intervention group, being hospitalized, number of admission diagnoses, waiting time, and total length of stay. The association between delirium and the previously mentioned independent variables was tested through a logit regression where delirium was dichotomized as previously described.

### Ethical Issues

The research protocol was approved by the Ethics and Scientific Committee of IMSS (R2011–785-056). Informed written consent was signed by the included participants or by their designated representative. Patients were free to refuse to take part in the study or to withdraw from it at any point; continued care was ensured.

## Results

**Figure 1:**
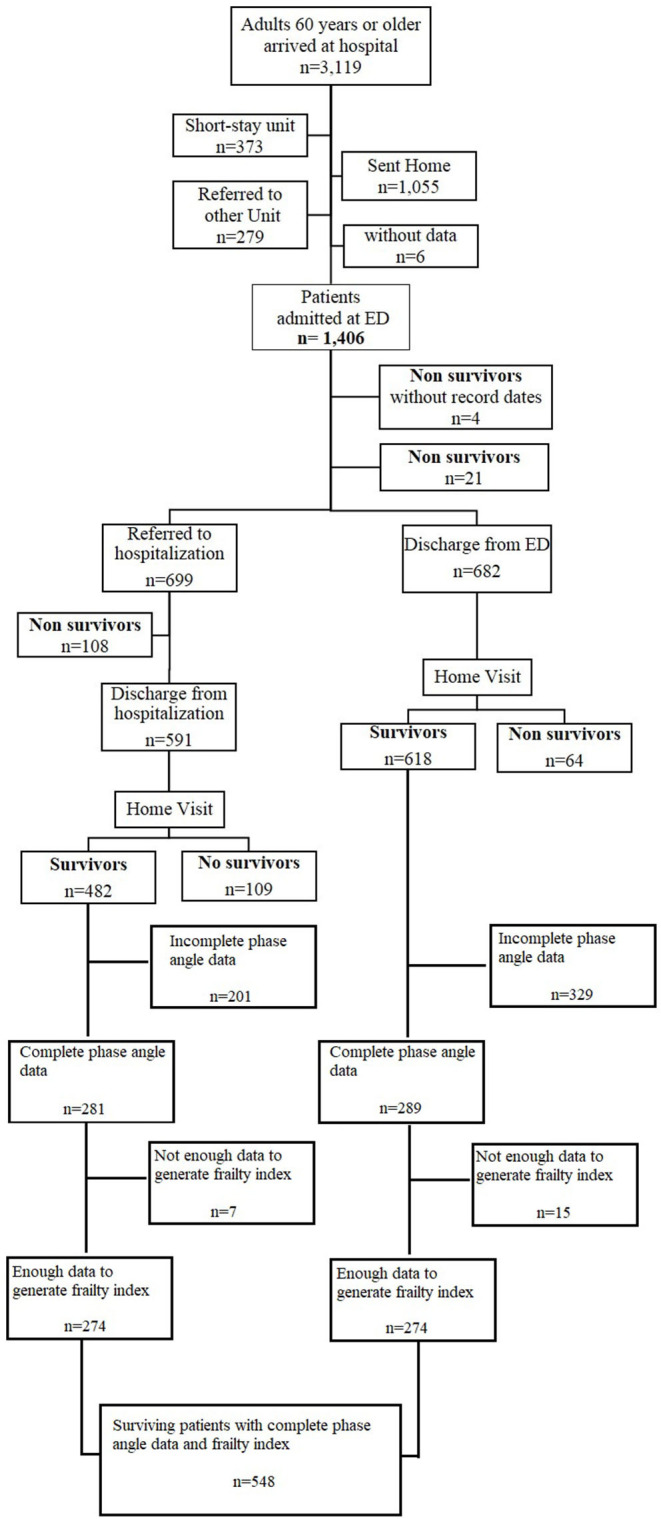
Flow chart.

During the study period a total of 3,119 patients 60 years and older arrived at the ED (Figure 1). Among the 1,100 who were alive post-discharge, 570 had phase angle data, and among these individuals, 548 had enough data to identify their FI level.

From this final sample, mean age was 76 (± standard deviation [SD] 7.5) and 58.4% (*n* = 320) were women. The average number of years in school was 7.3 (± SD 4.3 years), and 42.4% (*n* = 222) were married. Nearly 10% (*n* = 53) lived alone and a high percentage (58.9%, *n* = 305) perceived their financial situation as bad (Table 1).

**Table 1:** Sociodemographic characteristics of the studied individuals by frailty level.

	**Frailty at admission**				**Total** ***N* = 548**	***P*-value**
**Variables**	** <0.2** ***N* = 111**	**0.2–0.39** ***N* = 247**	**0.4–0.59** ***N* = 120**	**≥0.6** ***N* = 70**		
Sociodemographic						
Age, mean (SD)	75.9 (7.4)	75.3 (7.2)	76.6 (8.0)	77.4 (7.5)	76.0 (7.5)	0.1863
Sex, *n* (%)						
Man	54 (48.7)	106 (42.9)	39 (32.5)	29 (41.4)	228 (41.6)	0.0880^*^
Woman	57 (51.4)	141 (57.1)	81 (67.5)	41 (58.6)	320 (58.4)	
Schooling, mean (SD)	8.4 (4.4)	7.6 (4.2)	6.4 (3.9)	5.9 (4.5)	7.3 (4.3)	0.0010^***^
Civil status, *n* (%)						
Married	59 (55.7)	102 (43.8)	41 (35.3)	20 (29.0)	222 (42.4)	0.0000^***^
Widower	36 (34.0)	95 (40.8)	66 (56.9)	32 (46.4)	229 (46.4)	
Other	11 (10.4)	36 (15.5)	9 (7.8)	17 (24.6)	73 (13.9)	
Lives alone, *n* (%)	14 (12.7)	17 (6.9)	13 (10.8)	9 (12.9)	53 (9.7)	0.2250
Self-perception Financial situation, *n* (%)						
Good	55 (51.4)	96 (41.0)	47 (41.2)	15 (23.8)	213 (41.1)	0.0060^***^
Bad	52 (48.6)	138 (59.0)	67 (58.8)	48 (76.2)	305 (58.9)	
Health service						
Waiting time (in hours) in the ER, mean (SD)	5.6 (7.8)	5.0 (5.5)	5.1 (5.9)	5.1 (6.3)	5.1 (6.2)	0.5115
Length of stay (in hours) in ER, mean (SD)	87.7 (69.7)	93.6 (69.3)	112.0 (63.7)	121.5 (63.8)	99.9 (68.2)	0.0022^***^
Total length of stay (in hours), mean (SD)	113.5 (87.3)	144.6 (116.8)	155.5 (100.6)	172.7 (102.3)	144.1 (107.2)	0.0399^**^
Geriatric care trained residents, *n* (%)						
Yes	26 (23.4)	60 (24.2)	33 (27.5)	21 (30.0)	140 (25.6)	0.6970
No	85 (76.6)	187 (75.7)	87 (72.5)	49 (70.0)	408 (74.5)	
Referral to hospitalization, *n* (%)						
Yes	54 (48.7)	123 (49.8)	58 (58.3)	39 (55.7)	274 (50.0)	0.7690
No	57 (51.4)	124 (50.2)	62 (51.7)	31 (44.3)	274 (50.0)	
Health						
Admission reason, *n* (%)						
Anorexia/weight lost	0 (0.0)	2 (0.8)	1 (0.8)	0 (0.0)	3 (0.6)	0.1100
Neurological problems	8 (7.2)	11 (4.5)	8 (6.7)	4 (5.7)	31 (5.7)	
Associated diabetes problems	1 (0.9)	7 (2.8)	2 (1.7)	0 (0.0)	10 (1.8)	
Cardiovascular problems	10 (9.0)	16 (6.5)	5 (4.2)	2 (2.9)	33 6.0)	
Pneumological problems	7 (6.3)	21 (8.5)	10 (8.3)	3 (4.3)	41 (7.5)	
Gastrointestinal problems	22 (19.8)	38 (15.4)	30 (25.0)	6 (8.6)	96 (17.5)	
Genitourinary problems	7 (6.3)	10 (4.1)	2 (1.7)	3 (4.3)	22 (4.0)	
Non-specific symptoms	14 (12.6)	33 (13.4)	17 (14.2)	9 (12.9)	73 (13.3)	
Procedures/other services	2 (1.8)	9 (3.6)	3 (2.5)	2 (2.9)	16 (2.9)	
Other reasons	8 (7.2)	4 (1.6)	6 (5.0)	6 (8.6)	24 (4.3)	
Not specified	32 (28.8)	96 (38.9)	36 (30.0)	35 (50.0)	199 (36.3)	
Number of admission reasons, median (RIC)	2 (1)	2 (2)	2 (2)	2 (1)	2 (2)	0.0011^***^
Delirium scale changes, *n* (%)						
Remained without delirium or lost it	104 (100)	227 (96.6)	106 (93.8)	53 (76.8)	490 (94.1)	0.0000^***^
Remained with delirium or acquired it	0 (0.0)	8 (3.4)	7 (6.2)	16 (23.2)	31 (6.0)	
Phase Angle, mean (SD)						
Phase Angle—Admission to ER	5.3 (1.5)	5.3 (1.9)	5.0 (1.9)	4.7 (1.7)	5.2 (1.8)	0.0023^***^
Phase Angle—Discharge from ER	5.4 (1.8)	5.2 (1.7)	5.1 (2.9)	4.8 (1.5)	5.2 (2.0)	0.0168^**^
Phase Angle—Discharge from hospitalization	5.1 (1.7)	5.5 (1.9)	4.8 (1.8)	5.9 (5.5)	5.3 (2.7)	0.2227
Phase Angle—Home evaluation	5.6 (1.9)	5.5 (2.3)	5.4 (3.3)	5.3 (2.7)	5.5 (2.5)	0.0540^*^
Hand grip strength, mean (SD)						
Hand grip strength—Admission to ER	8.5 (9.1)	5.5 (7.2)	2.6 (4.1)	1.8 (4.3)	5.0 (7.2)	0.0001^***^
Hand grip strength—Discharge from ER	11.5 (8.8)	7.5 (7.9)	3.4 (4.5)	3.0 (6.6)	6.9 (7.9)	0.0001^***^
Hand grip strength—Discharge from hospitalization	13.4 (7.4)	7.7 (7.1)	2.7 (3.6)	2.4 (4.0)	6.8 (7.2)	0.0001^***^
Hand grip strength—Home evaluation	14.5 (9.8)	12.3 (9.4)	9.8 (8.2)	10.4 (9.2)	11.9 (9.3)	0.0004^***^

* P value < 0.1,

** P value < 0.05,

*** *P value < 001*.

Regarding health care process variables, the mean waiting time in the ED was 5.1 h (± SD 6.2), the average stay in the ED was 99.9 (±68.2) h, and 274 subjects (50%) were admitted to a general ward (Table 1). According to FI, these same variables had a length of stay in ED was 87.7 h (± SD 69.7) for FI <0.2, 93.6 h (± SD 69.3) for FI=0.2 to 0.39, 112 h (± SD 63.7) for FI=0.4 to 0.59, and 121.5 h (± SD 63.8) for FI greater or equal to 0.6 (*p* = 0.0022).

Delirium (*p* < 0.001), phase angle at admission to ED (*p* = 0.002), and handgrip strength at all stages (*p* < 0.01) were different across levels of frailty. Significant differences (*p* = 0.03) were also found in total length of stay (*p* = 0.04) and phase angle at discharge from ED (*p* = 0.02) (Table 1). FI was negatively associated with phase angle, but this association was not significant at any stage (Table 2). Phase angle at admission to ED was associated with sex (*p* = 0.02), living alone (*p* = 0.02), and age (*p* = 0.01), while sex was associated with phase angle at the home visit (*p* = 0.04) (Supplementary Table 2).

**Table 2:** Association between phase angle and hand grip strength with frailty.

		**Admission at ER**					**Discharge at ER**					**Discharge of hospitalization**					**Visit at home**				
			**95% CI**					**95% CI**					**95% CI**					**95% CI**			
		**β**	**Lower**	**Upper**	**Standard β**	***P***	**β**	**Lower**	**Upper**	**Standard β**	***P***	**β**	**Lower**	**Upper**	**Standard β**	***P***	**β**	**Lower**	**Upper**	**Standard ß**	***P***
Phase angle	Frailty category																				
	≥0.4	−0.38	−0.87	0.10	0.25	0.12	−0.10	−0.51	0.30	0.20	0.61	−0.80	−1.93	0.33	0.57	0.16	−0.48	−1.09	0.12	0.31	0.12
Hand grip strength	Frailty category																				
	≥0.4	−3.97	−5.56	−2.38	0.81	0.00	−3.94	−5.97	−1.90	1.03	0.00	−4.93	−7.68	−2.18	1.38	0.00	−1.41	−3.13	0.30	0.87	0.11

*Values adjusted using the following variables: marital status, education level, sex, living alone, economic situation, intervention, being hospitalized, age, number of admission reasons, waiting time in the ED and length of stay*.

FI was negatively associated with handgrip strength at admission to ED (*p* < 0.001), discharge from ED (*p* < 0.001), and discharge from hospitalization (*p* = 0.01) (Table 2). Other variables associated with handgrip strength were being a widow (*p* < 0.001), having a bachelor's degree or higher (*p* = 0.03), and sex (*p* < 0.01) (Supplementary Table 3). At the home visit, sex (*p* < 0.01), intervention (*p* = 0.02), and age (*p* < 0.01) were associated with grip strength. Finally, the FI was found to be positively associated with worsened delirium *p* = 0.001) (Table 3). The only other variable that was associated with delirium was the intervention (*p* = 0.01).

**Table 3:** Association between delirium with frailty and other variables.

	**Worse delirium between admission at ER and discharge at ER**					**Worse delirium between admission at ER and discharge at ER (OR)**				
		**95% CI**					**95% CI**			
	**β**	**lower**	**upper**	**Standard β**	***P***	**OR**	**lower**	**upper**	**Standard Error**	***P***
Frailty category										
≥0.4	3.68	1.53	5.83	1.10	0.00	**39.59**	**4.63**	**338.69**	**43.36**	**0.00**
Marital status										
Widower	−0.27	−1.72	1.17	0.74	0.71	0.76	0.18	3.21	0.56	0.71
Other	0.12	−1.99	2.23	1.08	0.91	1.13	0.14	9.32	1.22	0.91
Education level										
Secondary or high school	−0.19	−1.67	1.30	0.76	0.81	0.83	0.19	3.66	0.63	0.81
Bachelor and more	0.24	−2.52	3.00	1.41	0.86	1.28	0.08	20.17	1.80	0.86
Woman	−1.20	−2.43	0.03	0.63	0.06	0.30	0.09	1.03	0.19	0.06
Bad economic situation	0.45	−0.84	1.73	0.66	0.50	1.56	0.43	5.65	1.03	0.50
Intervention	2.00	0.52	3.48	0.75	0.01	**7.38**	**1.69**	**32.33**	**5.56**	**0.01**
Age	0.00	−0.08	0.09	0.04	0.96	1.00	0.92	1.09	0.04	0.96
Number of admission reasons	0.25	−0.23	0.73	0.24	0.30	1.29	0.80	2.08	0.31	0.30
Waiting time in the ED	0.04	−0.06	0.14	0.05	0.39	1.04	0.95	1.15	0.05	0.39
Length of stay	0.00	−0.00	0.01	0.00	0.70	1.00	1.00	1.01	0.00	0.70

*Bold values represent the only variables that were associated with worsened delirium (p < 0.05)*.

## Discussion

### Main Findings

Frailty in older adults attending the ED is a relatively novel topic, and our study aids in narrowing some gaps about this issue. Moreover, it also reports on how frailty levels impact the presence of these complications, further burdening the health status of the individuals and increasing the risk of adverse health outcomes. Nearly 35% of the patients had frailty levels greater or equal to 0.4 at admission to ED. Hand grip strength was significantly associated with frailty in almost all stages of hospitalization but not at post-discharge home evaluation. Worse delirium was also significantly related to frailty levels. In the case of phase angle, the associations were not statistically significant.

It is well-established that frailty represents a vulnerable state that results in worse health status when frail people face stressors (28). Previous work has shown that Mexican older adults have high levels of frailty (25). Our study shows that an already frail older adult who is hospitalized is at the highest risk of developing adverse outcomes. In the previous work of this hospitalized cohort, we reported a high mortality rate (21.8%) (24). Moreover, more than half of the participants had frailty levels above 0.2. This benchmark is generally considered to be overt frailty and is associated with mortality in Mexican older adults (29).

A systematic review by Goldstein et al. reported scarce evidence on frailty in the ED, however the interest in this topic seems to be growing (30). Afilalo et al. recently reported on a prospective cohort study on persons 75 years or older discharged from the ED (31). They used gait speed and grip strength as markers of frailty and other adverse outcomes. Their results demonstrated that only gait speed was a predictor of frailty before ED discharge. Additionally, diminished gait speed is a risk factor for functional decline (OR 1.4, 95% CI 1.1–1.7) and subsequent ED visits.

Moreover, Joosten et al. reported the predictive validity of frailty, measured using the phenotype and the Study of Osteoporotic Fracture tool in patients admitted to an acute geriatric ward (32). A total of 511 patients were selected but 250 were excluded, and 40% of individuals were classified as frail 24 h prior to admission. Frailty was not significantly associated with delirium, even after taking into account that 95% of participants were referred from the ED. In contrast, Eeles et al. (33) studied 273 patients admitted to a general medical service. Using a 33-item FI, they found that patients with delirium had significantly higher FI scores.

Contrary to what we expected, phase angle was not associated with frailty. Our hypothesis was supported by a recent report by Zanforlini et al. where phase angle predicted frailty levels over time in a sample of fit community-dwelling older people. More studies need to investigate the possible role of phase angle as a predictive tool for complications in older adults entering the ED (34).

Our results show that the frailer an individual is, the higher the probability of having adverse outcomes. This may be explained by a competing risk phenomenon (35). Moreover, there are limited geriatric care resources, which may account for the high rate of hospital complications, since early screening for hospitalized older adults are not routinely conducted (36). Our results could be similar to other contexts, since EDs worldwide are busy and unprepared to treat the oldest patients (15). On the other hand, our sample has unique features that could differentiate results, such as high rates of diabetes, hypertension, and overall metabolic syndrome (2, 17).

### Limitations

One of the main limitations of our work is that there is no consensus on how to define ED complications; therefore, our study should be considered exploratory. However, it addresses the growing impact of frailty on health care systems and the further deterioration experienced by older adults (9, 24, 37). That being said, the high number of excluded individuals may be responsible for the non-significant associations of phase angle with frailty. However, this situation is typical in ED studies for older adults, where data collection is already a challenge (38). As ED services are highly heterogeneous, this might advise the reader to interpret our results in light of the context. In the same vein, local features of the Mexican health system merit a cautious interpretation of the data. Many frailty experts advise against the categorization of the FI but we used these arbitrary groups for a better understanding of the burden depicted into the FI.

### Conclusion

Frailty is associated with geriatric conditions in Mexican older adults attending the ED.

## Data Availability Statement

The raw data supporting the conclusions of this article will be made available by the authors, without undue reservation.

## Ethics Statement

The studies involving human participants were reviewed and approved by Ethics and Scientific Committee of IMSS (R2011–785-056). The patients/participants provided their written informed consent to participate in this study. Written informed consent was obtained from the individual(s) for the publication of any potentially identifiable images or data included in this article.

## Author Contributions

MP-Z: concept of the study, analysis interpretation of data, and preparation of the first draft. MC-V: concept of the study, interpretation of data, and preparation of the manuscript. LJ-M: analysis and interpretation of data, and preparation of manuscript. OT: revised the first draft. CG-P: concept and design of the study, acquisition of subjects and funding, interpretation of data, and preparation of the manuscript. All authors contributed to the article and approved the submitted version.

## Conflict of Interest

The authors declare that the research was conducted in the absence of any commercial or financial relationships that could be construed as a potential conflict of interest.
