# Supplementary material for: Hospital Complications and Frailty in Mexican Older Adults: An Emergency Care Cohort Analysis
Source: Front Med (Lausanne). 2020 Sep 29;7:505. doi: 10.3389/fmed.2020.00505 (PMC7550456; doi:10.3389/fmed.2020.00505)
Supplement: Supplementary file 1 [file Table_1.DOCX]

Supplementary table 1. Complete items included and coding of the Frailty index

| Variable | Description | Coding for the FI | Frequency or mean | |
| --- | --- | --- | --- | --- |
|  |  |  | Baseline | Follow-up |
| Self-rated vision | How would you rate your vision? | 1 = Legally blind  0.8 = Poor  0.6 = Fair  0.4 = Good  0.2 = Very good  0 = Excellent | 1.55  14.23  36.06  43.44  4.2  0.52 | 1.42  19.43  42.25  32.31  3.49  1.09 |
| Falls | Have you fallen in the last two years? | 1 = Yes  0 = No | 55.74  44.26 | 23.57  76.43 |
| Hearing aid | Do you use a hearing aid? | 1 = Yes  0 = No | 6.69  93.31 | 5.29  94.71 |
| Pain | How much pain do you have? | 1 = Severe  0.5 = Moderate  0 = None | 18.4  51.36  30.24 | 14.67  39.95  45.74 |
| Depression | Are you depressed? | 1 = Very much  0.5 = Much  1 = No | 19.42  47.8  32.78 | 16.11  43.57  40.32 |
| Fatigue | In the last four weeks, how much of the time have you felt fatigued? | 1 = All the time  0.75 = Most of the time  0.5 = Sometimes  0.25 = Few times  0 = Never | 25.73  30.5  21.33  15.29  7.16 | 16.07  23.67  19.65  23.02  17.59 |
| Weight loss | Have you lost weight in the last three months? | 1 = Lost ≥3kg  0.5 = Lost 1-2.9kg  0 = No | 22.84  37.1  40.06 | 22.65  27.4  49.95 |
| Respiratory symptoms | In the last two years, have you had cough, phlegm or dyspnea? | 1 = Yes  0 = No | 48.89  51.11 | 47.19  52.81 |
| Gastrointestinal symptoms | In the last two years, have you had stomach pain, indigestion or diarrhea? | 1 = Yes  0 = No | 58.75  41.25 | 39.85  60.15 |
| Urinary incontinence | In the last two years, have you had urinary involuntary loss? | 1 = Yes  0 = No | 39.87  60.13 | 36.37  63.63 |
| Mental orientation | Mini-mental status examination questions on orientation | Inverse reciprocal of the maximum score (10 | 0.342 | 0.155 |
| Walking | Do you have any difficulty to walk up to 10 steps without stopping? | 1 = Yes  0 = No | 43.45  56.55 | 36.78  63.22 |
| Blocks | Do you have any difficulty to walk up to 100 meters by yourself? | 1 = Yes  0 = No | 67.73  32.27 | 61.48  38.52 |
| Moving in bed | Do you have any difficulty to move in or out of your bed? | 1 = Yes  0 = No | 27.51  72.49 | 28.4  71.6 |
| Climbing | Are you capable of climbing one flight of stairs by yourself? | 1 = Yes  0 = No | 43.32  56.68 | 52.65  47.35 |
| Picking up | Do you have any difficulty to pick up a coin from bed? | 1 = Yes  0 = No | 27.73  72.27 | 22.51  77.49 |
| Dressing | Do you have any difficulty to dress yourself, including shoes and socks? | 1 = Yes  0 = No | 33.18  66.82 | 33.74  66.26 |
| Room moving | Do you have any difficulty to move into your room? | 1 = Yes  0 = No | 23.32  76.68 | 24.75  75.25 |
| Bathing | Do you have any difficulty to take a bath on the shower or the tub? | 1 = Yes  0 = No | 33.16  66.84 | 32.38  67.62 |
| Eating | Do you have any difficulty to eat, for example, cutting with a knife your food? | 1 = Yes  0 = No | 19.3  80.7 | 19.98  80.02 |
| Toileting | Do you have any difficulty to use the toilet? | 1 = Yes  0 = No | 24.28  75.72 | 23.4  76.6 |
| Taking medicine | Do you have any difficulty to take your medicines? | 1 = Yes  0 = No | 24.7  75.3 | 20.83  79.17 |
| Anorexia | In the last three months, have you eaten less because of loss of appetite, chewing or swallowing problems? | 1 = Severe  0.5 = Moderate  0 = None | 17  37.47  45.53 | 11.02  26.57  62.42 |
| Limb pain | In the last two years, have you had pain in your legs after walking? | 1 = Yes  0 = No | 65.57  34.43 | 61.42  38.58 |
| Bowel incontinence | Do you have bowel incontinence | 1 = Yes  0.5 = Sporadic  0 = No | 10.02  17.48  72.49 | 8.96  16.2  74.84 |
| Effort | In the last week, how frequently have you felt that everything you do requires an effort? | 1 = Always  0.6 = Frequently  0.3 = Occasionally  0 = Never | 31.73  24.16  17.03  27.08 | 28.79  25.76  18.61  26.84 |
| Hypertension | Has a doctor told you that you have hypertension? | 1 = Yes  0 = No | 66.94  33.06 | 68.57  31.43 |
| Cancer | Has a doctor told you that you have cancer? | 1 = Yes  0 = No | 8.02  91.98 | 8.12  91.88 |
| Chronic obstructive pulmonary disease | Has a doctor told you that you have chronic obstructive pulmonary disease? | 1 = Yes  0 = No | 15.21  84.79 | 16.96  83.04 |
| Stroke | Has a doctor told you that you have had a stroke? | 1 = Yes  0 = No | 10.39  89.61 | 12.55  87.45 |
| Arthritis | Has a doctor told you that you have arthritis? | 1 = Yes  0 = No | 15.16  84.84 | 13.03  86.97 |
| Could not go on | In the last week, how frequently have you felt that you could not go on? | 1 = Always  0.6 = Frequently  0.3 = Occasionaly  0 = Never | 24.16  23.56  19.05  33.23 | 21.91  22.23  19.31  36.55 |

| Supplementary table 2. Association between phase angle with frailty and other variables | | | | | | | | | | | | | | | | | | | | |
| --- | --- | --- | --- | --- | --- | --- | --- | --- | --- | --- | --- | --- | --- | --- | --- | --- | --- | --- | --- | --- |
|  | Phase angle at admission at ER | | | | | Phase angle at discharge at ER | | | | | Phase angle at discharge of hospitalization | | | | | Phase angle at visit at home | | | | |
|  |  | 95% CI | |  |  |  | 95% CI | |  |  |  | 95% CI | |  |  |  | 95% CI | |  |  |
|  | β | lower | upper | Standard β | P | β | lower | upper | Standard β | P | β | lower | upper | Standard β | P | β | lower | upper | Standard ß | P |
| **Marital status** |  |  |  |  |  |  |  |  |  |  |  |  |  |  |  |  |  |  |  |  |
| Widower | 0.03 | -0.48 | 0.54 | 0.26 | 0.91 | -0.35 | -0.77 | 0.08 | 0.22 | 0.11 | -1.19 | -2.36 | -0.03 | 0.58 | 0.05 | 0.35 | -0.29 | 1.00 | 0.33 | 0.28 |
| Other | 0.06 | -0.67 | 0.80 | 0.37 | 0.87 | -0.37 | -0.98 | 0.25 | 0.31 | 0.24 | -0.73 | -2.93 | 1.47 | 1.10 | 0.51 | 0.52 | -0.40 | 1.44 | 0.47 | 0.27 |
| **Education level** |  |  |  |  |  |  |  |  |  |  |  |  |  |  |  |  |  |  |  |  |
| Secondary or high school | 0.29 | -0.25 | 0.83 | 0.28 | 0.29 | -0.18 | -0.64 | 0.27 | 0.23 | 0.42 | -1.09 | -2.37 | 0.20 | 0.64 | 0.10 | 0.13 | -0.55 | 0.81 | 0.35 | 0.71 |
| Bachelor and more | -0.85 | -1.68 | -0.02 | 0.42 | 0.05 | -0.39 | -1.09 | 0.30 | 0.35 | 0.27 | 0.25 | -2.18 | 2.67 | 1.21 | 0.84 | -0.67 | -1.67 | 0.42 | 0.53 | 0.24 |
| Woman | -0.57 | -1.05 | -0.09 | 0.24 | 0.02 | -0.22 | -0.61 | 0.18 | 0.20 | 0.28 | -0.26 | -1.37 | 0.85 | 0.55 | 0.64 | -0.63 | -1.22 | -0.03 | 0.30 | 0.04 |
| Living alone | -0.97 | -1.78 | -0.17 | 0.41 | 0.02 | -0.49 | -1.16 | 0.18 | 0.34 | 0.15 | 0.12 | -1.53 | 1.78 | 0.83 | 0.88 | -0.17 | -1.18 | 0.83 | 0.52 | 0.73 |
| Bad economic situation | -0.14 | -0.61 | 0.31 | 0.23 | 0.53 | -0.18 | -0.57 | 0.20 | 0.20 | 0.35 | 0.14 | -0.91 | 1.19 | 0.53 | 0.79 | 0.07 | -0.51 | 0.65 | 0.30 | 0.80 |
| Intervention | -0.08 | -0.70 | 0.55 | 0.32 | 0.81 | 0.15 | -0.37 | 0.67 | 0.26 | 0.57 | 1.39 | -0.20 | 2.99 | 0.80 | 0.09 | 0.68 | -0.10 | 1.46 | 0.40 | 0.09 |
| Being hospitalized | -0.23 | -0.86 | 0.40 | 0.32 | 0.47 | -0.14 | -0.69 | 0.38 | 0.27 | 0.60 | - | - | - | - | - | -0.77 | -1.56 | 0.02 | 0.40 | 0.06 |
| Age | -0.05 | -0.08 | -0.02 | 0.02 | 0.00 | -0.04 | -0.06 | -0.01 | 0.01 | 0.01 | 0.06 | -0.02 | 0.13 | 0.04 | 0.12 | -0.04 | -0.08 | -0.00 | 0.02 | 0.05 |
| Number of admission reasons | 0.10 | -0.09 | 0.29 | 0.10 | 0.31 | -0.01 | -0.17 | 0.15 | 0.08 | 0.92 | 0.42 | -0.13 | 0.98 | 0.28 | 0.13 | -0.08 | -0.32 | 0.16 | 0.13 | 0.51 |
| Waiting time in the ED | -0.03 | -0.06 | 0.01 | 0.02 | 0.16 | -0.02 | -0.05 | 0.01 | 0.02 | 0.21 | 0.00 | -0.06 | 0.07 | 0.03 | 0.97 | 0.02 | -0.02 | 0.07 | 0.02 | 0.32 |
| Length of stay | -0.00 | -0.00 | 0.00 | 0.00 | 0.23 | -0.00 | -0.01 | -0.00 | 0.00 | 0.02 | -0.00 | -0.01 | 0.00 | 0.00 | 0.18 | 0.00 | -0.00 | 0.00 | 0.00 | 0.58 |

Supplementary table 3. Association between hand grip strength with frailty and other variables

|  | Hand grip strength at admission at ER | | | | | Hand grip strength at discharge at ER | | | | | Hand grip strength at discharge of hospitalization | | | | | Hand grip strength at visit at home | | | | |
| --- | --- | --- | --- | --- | --- | --- | --- | --- | --- | --- | --- | --- | --- | --- | --- | --- | --- | --- | --- | --- |
|  |  | 95% CI | |  |  |  | 95% CI | |  |  |  | 95% CI | |  |  |  | 95% CI | |  |  |
|  | β | lower | upper | Standard β | P | β | lower | upper | Standard β | P | β | lower | upper | Standard β | P | β | lower | upper | Standard ß | P |
| **Marital status** |  |  |  |  |  |  |  |  |  |  |  |  |  |  |  |  |  |  |  |  |
| Widower | -2.71 | -4.40 | -1.02 | 0.86 | 0.00 | -3.15 | -5.24 | -1.06 | 1.06 | 0.00 | -2.36 | -5.23 | 0.51 | 1.44 | 0.11 | -1.02 | -2.84 | 0.81 | 0.93 | 0.27 |
| Other | -2.38 | -4.79 | 0.04 | 1.23 | 0.05 | -2.84 | -5.91 | 0.23 | 1.56 | 0.07 | 5.08 | -0.33 | 9.83 | 2.38 | 0.04 | 0.13 | -2.47 | 2.73 | 1.32 | 0.92 |
| **Education level** |  |  |  |  |  |  |  |  |  |  |  |  |  |  |  |  |  |  |  |  |
| Secondary or high school | -0.48 | -2.27 | 1.31 | 0.91 | 0.60 | 0.20 | -2.02 | 2.42 | 1.13 | 0.86 | 0.79 | -2.26 | 3.84 | 1.53 | 0.61 | 0.33 | -1.63 | 2.30 | 1.00 | 0.74 |
| Bachelor and more | 3.02 | 0.28 | 5.76 | 1.39 | 0.03 | 3.85 | 0.19 | 7.52 | 1.86 | 0.04 | -3.11 | -10.2 | 3.93 | 3.53 | 0.38 | 0.47 | -2.57 | 3.50 | 1.54 | 0.76 |
| Woman | -3.76 | -5.33 | -2.20 | 0.80 | 0.00 | -4.38 | -6.34 | -2.43 | 0.99 | 0.00 | -4.63 | -7.31 | -1.95 | 1.34 | 0.00 | -11.2 | -12.9 | -9.51 | 0.86 | 0.00 |
| Living alone | -1.42 | -4.07 | 1.23 | 1.35 | 0.29 | -0.73 | -3.97 | 2.51 | 1.64 | 0.67 | -3.20 | -7.32 | 0.92 | 2.06 | 0.13 | 0.65 | -2.27 | 3.50 | 1.48 | 0.66 |
| Bad economic situation | -1.13 | -2.65 | 0.39 | 0.77 | 0.15 | -0.83 | -2.78 | 1.13 | 0.99 | 0.41 | -1.88 | -4.44 | 0.68 | 1.28 | 0.15 | -0.89 | -2.54 | 0.77 | 0.84 | 0.29 |
| Intervention | 1.33 | -0.72 | 3.38 | 1.04 | 0.20 | 1.51 | -1.26 | 4.28 | 1.41 | 0.28 | 2.61 | -1.50 | 6.73 | 2.06 | 0.21 | 2.67 | 0.46 | 4.88 | 1.12 | 0.02 |
| Being hospitalized | -0.11 | -2.20 | 1.97 | 1.06 | 0.91 | 0.39 | -2.25 | 3.03 | 1.34 | 0.77 | - | - | - | - | - | -1.58 | -3.83 | 0.67 | 1.14 | 0.17 |
| Age | -0.02 | -0.13 | 0.08 | 0.05 | 0.63 | -0.07 | -0.20 | 0.06 | 0.06 | 0.30 | -0.06 | -0.23 | 0.12 | 0.09 | 0.53 | -0.30 | -0.41 | -0.19 | 0.06 | 0.00 |
| Number of admission reasons | -0.29 | -0.93 | 0.35 | 0.33 | 0.38 | -0.25 | -1.03 | 0.52 | 0.39 | 0.52 | 0.57 | -0.79 | 1.92 | 0.68 | 0.41 | -0.30 | -1.00 | 0.39 | 0.35 | 0.39 |
| Waiting time in the ED | -0.05 | -0.17 | 0.07 | 0.06 | 0.45 | -0.03 | -0.17 | 0.11 | 0.07 | 0.66 | -0.06 | -0.27 | 0.14 | 0.10 | 0.54 | -0.00 | -0.13 | 0.13 | 0.07 | 0.97 |
| Length of stay | -0.01 | -0.02 | 0.00 | 0.00 | 0.16 | -0.01 | -0.02 | 0.01 | 0.01 | 0.19 | -0.01 | -0.02 | 0.01 | 0.01 | 0.40 | 0.00 | -0.01 | 0.01 | 0.01 | 0.78 |
